# (2,3,7,8,12,13,17,18-Octa­ethyl-5-phenyl­porphyrinato)platinum(II)

**DOI:** 10.1107/S1600536811026882

**Published:** 2011-07-13

**Authors:** Mathias O. Senge, Julia Richter

**Affiliations:** a School of Chemistry, SFI Tetrapyrrole Laboratory, Trinity College Dublin, Dublin 2, Ireland

## Abstract

The title compound, [Pt(C_42_H_48_N_4_)], was obtained through metallation of the corresponding free base with PtCl_2_, followed by crystallization from methyl­ene chloride/methanol. The mol­ecule exhibits an almost planar macrocycle with an average deviation of the 24 macrocyclic atoms from their least-squares plane (Δ24) of 0.04 Å and an average Pt—N bond length of 2.022 Å. Despite the unsymmetrical substitution pattern, there is no significant difference between distortion of the geometry at the phenyl substituted *meso* position and those of unsubstituted *meso* positions.

## Related literature

For background to the conformation of porphyrins, see: Senge (2006 ▶); Senge *et al.* (1992 ▶, 2000 ▶). For the chemistry of highly substituted platinum(II) porphyrins with mixed *meso* substituents, see: Senge *et al.* (2010 ▶). For Pt(II) porphyrin structures, see: Hazell (1984 ▶); Milgrom *et al.* (1988 ▶); Senge (2000 ▶); Shmilovits *et al.* (2003 ▶); Umemiya *et al.* (2003 ▶). For handling of the crystals, see: Hope (1994 ▶). For details on normal-coord­i­nate structural decomposition analysis, see Jentzen *et al.* (1997 ▶).
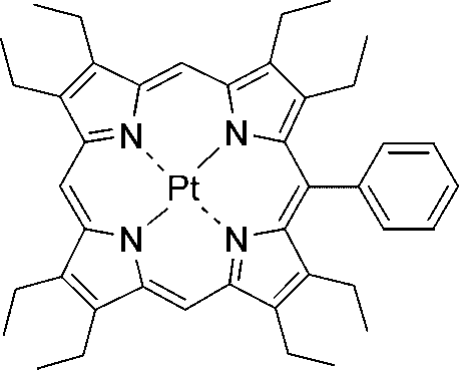


## Experimental

### 

#### Crystal data

[Pt(C_42_H_48_N_4_)]
*M* *_r_* = 803.93
Monoclinic, 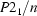

*a* = 17.1661 (6) Å
*b* = 8.9301 (3) Å
*c* = 22.8471 (8) Å
β = 99.367 (1)°
*V* = 3455.6 (2) Å^3^
*Z* = 4
Mo *K*α radiation
μ = 4.10 mm^−1^
*T* = 90 K
0.50 × 0.10 × 0.10 mm

#### Data collection

Bruker SMART CCD area-detector diffractometer
Absorption correction: multi-scan (*SADABS*; Bruker, 2004 ▶) *T*_min_ = 0.227, *T*_max_ = 0.664
43758 measured reflections
7989 independent reflections
6809 reflections with *I* > 2σ(*I*)
*R*_int_ = 0.037

#### Refinement

*R*[*F* ^2^ > 2σ(*F* ^2^)] = 0.020
*wR*(*F* ^2^) = 0.053
*S* = 1.06
7989 reflections
432 parameters
H-atom parameters constrained
Δρ_max_ = 1.15 e Å^−3^
Δρ_min_ = −0.60 e Å^−3^

### 

Data collection: *SMART* (Bruker, 2004 ▶); cell refinement: *SAINT-Plus* (Bruker, 2004 ▶); data reduction: *SAINT-Plus*; program(s) used to solve structure: *SIR92* (Altomare *et al.*, 1994 ▶); program(s) used to refine structure: *SHELXL97* (Sheldrick, 2008 ▶); molecular graphics: *SHELXTL* (Sheldrick, 2008 ▶); software used to prepare material for publication: *SHELXTL*.

## Supplementary Material

Crystal structure: contains datablock(s) global, I. DOI: 10.1107/S1600536811026882/ya2143sup1.cif

Structure factors: contains datablock(s) I. DOI: 10.1107/S1600536811026882/ya2143Isup2.hkl

Additional supplementary materials:  crystallographic information; 3D view; checkCIF report

## Figures and Tables

**Fig. 1.**
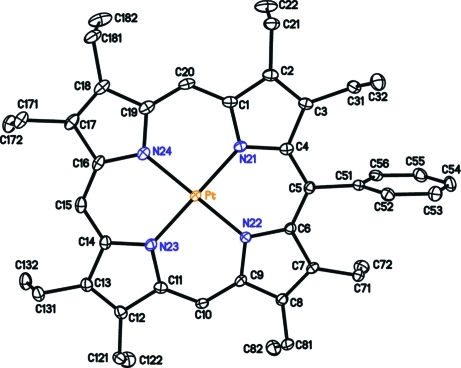
: Molecular structure of the title compound. Thermal ellipsoids are drawn at 50% probability level; hydrogen atoms have been omitted.

**Fig. 2.**
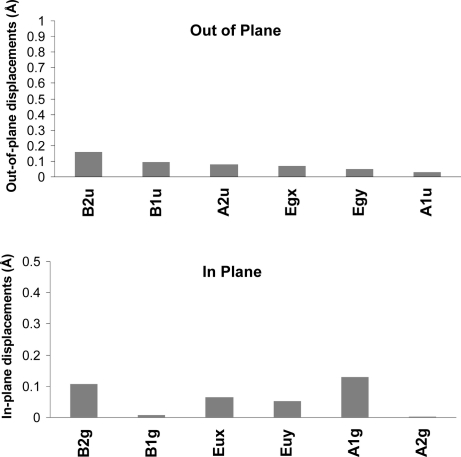
: Graphical representation of the displacements along the lowest-frequency coordinates that best simulate the porphyrin unit.

## supplementary crystallographic information

### Comment

In the context of our ongoing studies on the conformation of sterically hindered porphyrins (Senge, 2006) we have focused on the effect of *meso* phenyl substitution in the title compound. The compound was prepared through metallation of the corresponding free base and crystallized from CH_2_Cl_2_/CH_3_OH. The structure of the title compound is shown in Fig. 1. The molecule exhibits an almost planar macrocycle with an average deviation of 24 macrocyclic atoms from their least-squares plane (Δ24) of 0.04 Å and an average Pt–N bond length of 2.022 Å. Despite the unsymmetrical substitution pattern, there is no significant difference between distortion of the geometry at the C5 atom (carrying the phenyl residue) and those of the other *meso* positions. The low degree of conformational distortion is evidenced by a normal-coordinate structural decomposition (NSD) analysis (Jentzen *et al.*, 1997). NSD is a means to deconvolute the individual distortion modes and to evaluate their individual contributions to the macrocycle distortion. Fig. 2 shows the results of the NSD analysis and indicates that the contributions from the invididual distortion modes are minor and comparable to each other. Note, related free base porphyrins typically show evidence of localized distortion as a result of *peri*-interactions (Senge *et al.*, 1992). The phenyl ring is approximately orthogonal to the mean porphyrin plane forming the dihedral angle of 91.7 (2)° with the plane of the four N atoms.

### Experimental

The free base porphyrin (60 mg, 0.098 mmol) and 52 mg PtCl_2_ (0.196 mmol, 2 equivalents) were refluxed at 188 °C in benzonitrile under an argon atmosphere. Heating was continued for 5 days until TLC monitoring showed a single red-brown product spot. The solvent was evaporated by distillation under reduced pressure (2 to 4 mbar at 120 °C) and the residue dissolved in a small amount of dichloromethane and filtered through a silica gel frit eluting with CH_2_Cl_2_/*n*-hexane (1:2, *v*/*v*). Further purification was achieved on a silica gel column with CH_2_Cl_2_/*n*-hexane (1:3, *v*/*v*) to yield 60 mg of red-brown crystals after precipitation from dichloromethane/methanol (0.74 mmol, 76%). *M*. p. 253 °C; ^1^H NMR (400 MHz, CDCl_3_): *d*= 10.02 (s, 2H, 10-*H*, 20-*H*), 9.98 (s, 1H, 15-*H*), 8.19 (d, 2H, *J* = 6.9 Hz, phenyl-*H*), 7.80 (t, 1H, *J* = 7.9 Hz, phenyl-*H*), 7.67 (t, 1H, *J* = 7.6 Hz, phenyl-*H*), 4.01 (q, 8H, *J* = 7.6 Hz, C*H**_2_*CH_3_), 3.93 (q, 4H, *J* = 7.5 Hz, C*H**_2_*CH_3_), 1.91 (m, 12H, CH*_2_*C*H*_3_), 1.86 (t, 6H, *J* = 7.6 Hz, CH*_2_*C*H*_3_), 1.13 p.p.m. (t, 6H, *J* = 7.5 Hz, CH*_2_*C*H*_3_); ^13^C NMR (100 MHz, CDCl_3_): *d*= 18.2, 19.5, 21.3, 29.6, 97.9, 99.3, 126.3, 128.4, 133.2, 137.0, 138.0, 139.1, 140.7, 141.6, 141.7, 143.0 p.p.m.; UV/vis (CH_2_Cl_2_): *λ*_max_ (lg *ε*) = 386 (6.60), 504 (5.28), 539 nm (5.78).

### Refinement

Hydrogen atoms were placed geometrically (C-H 0.95 Å for aromatic, 0.99 Å for methylene, and 0.98 Å for methyl H atoms) and included in the refinement in riding model approximation  with *U*(H) set to 1.2*U*_eq_(C) [1.5U_eq_(C) for methyl H atoms]. The highest peak of residual electron density is at the distance of 0.852 Å from the Pt atom.

### Crystal data

**Table:**

[Pt(C_42_H_48_N_4_)]	*F*(000) = 1624
*M**_r_* = 803.93	*D*_x_ = 1.545 Mg m^−^^3^
Monoclinic, *P*2_1_/*n*	Mo *K*α radiation, λ = 0.71073 Å
Hall symbol: -P 2yn	Cell parameters from 9498 reflections
*a* = 17.1661 (6) Å	θ = 4.8–55.2°
*b* = 8.9301 (3) Å	µ = 4.10 mm^−^^1^
*c* = 22.8471 (8) Å	*T* = 90 K
β = 99.367 (1)°	Needle, red-brown
*V* = 3455.6 (2) Å^3^	0.50 × 0.10 × 0.10 mm
*Z* = 4	

### Data collection

**Table:**

Bruker SMART CCD area-detector diffractometer	7989 independent reflections
Radiation source: sealed tube	6809 reflections with *I* > 2σ(*I*)
graphite	*R*_int_ = 0.037
phi and ω scans	θ_max_ = 27.6°, θ_min_ = 1.4°
Absorption correction: multi-scan (*SADABS*; Bruker, 2004)	*h* = −22→22
*T*_min_ = 0.227, *T*_max_ = 0.664	*k* = −11→11
43758 measured reflections	*l* = −29→29

### Refinement

**Table:**

Refinement on *F*^2^	Primary atom site location: structure-invariant direct methods
Least-squares matrix: full	Secondary atom site location: difference Fourier map
*R*[*F*^2^ > 2σ(*F*^2^)] = 0.020	Hydrogen site location: inferred from neighbouring sites
*wR*(*F*^2^) = 0.053	H-atom parameters constrained
*S* = 1.06	*w* = 1/[σ^2^(*F*_o_^2^) + (0.0235*P*)^2^ + 3.9546*P*] where *P* = (*F*_o_^2^ + 2*F*_c_^2^)/3
7989 reflections	(Δ/σ)_max_ = 0.006
432 parameters	Δρ_max_ = 1.15 e Å^−^^3^
0 restraints	Δρ_min_ = −0.60 e Å^−^^3^

### Special details

**Table:**

Geometry. All e.s.d.'s (except the e.s.d. in the dihedral angle between two l.s. planes) are estimated using the full covariance matrix. The cell e.s.d.'s are taken into account individually in the estimation of e.s.d.'s in distances, angles and torsion angles; correlations between e.s.d.'s in cell parameters are only used when they are defined by crystal symmetry. An approximate (isotropic) treatment of cell e.s.d.'s is used for estimating e.s.d.'s involving l.s. planes.
Refinement. Refinement of *F*^2^ against ALL reflections. The weighted *R*-factor *wR* and goodness of fit *S* are based on *F*^2^, conventional *R*-factors *R* are based on *F*, with *F* set to zero for negative *F*^2^. The threshold expression of *F*^2^ > σ(*F*^2^) is used only for calculating *R*-factors(gt) *etc*. and is not relevant to the choice of reflections for refinement. *R*-factors based on *F*^2^ are statistically about twice as large as those based on *F*, and *R*- factors based on ALL data will be even larger. Higher thermal librational movement was observed for some ethyl side chain carbon atoms. The nonstandard crystal setting was choosen on the basis of systematic absences in *XPREP*.

### Fractional atomic coordinates and isotropic or equivalent isotropic displacement parameters (Å^2^)

**Table:**

	*x*	*y*	*z*	*U*_iso_*/*U*_eq_	
Pt	0.099751 (5)	0.192585 (10)	0.898825 (4)	0.01038 (4)	
N21	0.07362 (13)	0.3564 (2)	0.83785 (9)	0.0122 (4)	
N22	0.20559 (13)	0.2911 (2)	0.92502 (10)	0.0118 (4)	
N23	0.12560 (13)	0.0283 (2)	0.95988 (10)	0.0132 (4)	
N24	−0.00526 (12)	0.0906 (2)	0.87159 (10)	0.0137 (4)	
C1	0.00573 (15)	0.3621 (3)	0.79639 (12)	0.0139 (5)	
C2	0.00634 (16)	0.4929 (3)	0.75972 (12)	0.0150 (5)	
C3	0.07411 (15)	0.5693 (3)	0.77917 (11)	0.0135 (5)	
C4	0.11799 (15)	0.4813 (3)	0.82804 (11)	0.0124 (5)	
C5	0.19422 (15)	0.5097 (3)	0.85827 (11)	0.0122 (5)	
C6	0.23629 (15)	0.4187 (3)	0.90267 (11)	0.0119 (5)	
C7	0.31682 (15)	0.4437 (3)	0.93470 (11)	0.0124 (5)	
C8	0.33098 (16)	0.3339 (3)	0.97658 (12)	0.0141 (5)	
C9	0.26224 (15)	0.2396 (3)	0.97018 (12)	0.0128 (5)	
C10	0.25645 (15)	0.1147 (3)	1.00507 (12)	0.0139 (5)	
H10	0.2999	0.0954	1.0355	0.017*	
C11	0.19446 (15)	0.0155 (3)	1.00036 (11)	0.0136 (5)	
C12	0.19191 (16)	−0.1167 (3)	1.03684 (12)	0.0146 (5)	
C13	0.12096 (16)	−0.1833 (3)	1.01810 (12)	0.0142 (5)	
C14	0.07986 (15)	−0.0924 (3)	0.97043 (11)	0.0132 (5)	
C15	0.00550 (16)	−0.1219 (3)	0.94010 (12)	0.0156 (5)	
H15	−0.0210	−0.2074	0.9519	0.019*	
C16	−0.03400 (15)	−0.0374 (3)	0.89372 (12)	0.0147 (5)	
C17	−0.11039 (15)	−0.0758 (3)	0.85982 (12)	0.0169 (6)	
C18	−0.12670 (15)	0.0298 (3)	0.81659 (13)	0.0170 (6)	
C19	−0.06125 (15)	0.1341 (3)	0.82456 (12)	0.0155 (5)	
C20	−0.05565 (16)	0.2593 (3)	0.79034 (12)	0.0159 (5)	
H20	−0.0985	0.2773	0.7592	0.019*	
C21	−0.05598 (16)	0.5299 (3)	0.70760 (12)	0.0184 (6)	
H21A	−0.0569	0.6395	0.7011	0.022*	
H21B	−0.1083	0.4997	0.7166	0.022*	
C22	−0.04139 (19)	0.4511 (4)	0.65095 (13)	0.0296 (7)	
H22A	0.0095	0.4834	0.6411	0.044*	
H22B	−0.0837	0.4769	0.6183	0.044*	
H22C	−0.0406	0.3424	0.6571	0.044*	
C31	0.09262 (16)	0.7167 (3)	0.75170 (12)	0.0155 (5)	
H31A	0.0717	0.7145	0.7087	0.019*	
H31B	0.1506	0.7292	0.7565	0.019*	
C32	0.05705 (17)	0.8509 (3)	0.77993 (13)	0.0199 (6)	
H32A	−0.0003	0.8381	0.7760	0.030*	
H32B	0.0687	0.9431	0.7597	0.030*	
H32C	0.0800	0.8572	0.8220	0.030*	
C51	0.23459 (15)	0.6497 (3)	0.84322 (11)	0.0126 (5)	
C52	0.22558 (16)	0.7814 (3)	0.87416 (12)	0.0149 (5)	
H52	0.1925	0.7829	0.9037	0.018*	
C53	0.26508 (16)	0.9106 (3)	0.86175 (13)	0.0192 (6)	
H53	0.2588	1.0006	0.8827	0.023*	
C54	0.31375 (16)	0.9082 (3)	0.81881 (13)	0.0200 (6)	
H54	0.3409	0.9965	0.8106	0.024*	
C55	0.32303 (17)	0.7772 (3)	0.78763 (13)	0.0193 (6)	
H55	0.3564	0.7760	0.7583	0.023*	
C56	0.28319 (16)	0.6482 (3)	0.79960 (12)	0.0151 (5)	
H56	0.2889	0.5589	0.7781	0.018*	
C71	0.37968 (15)	0.5560 (3)	0.92519 (12)	0.0162 (5)	
H71A	0.4120	0.5809	0.9639	0.019*	
H71B	0.3540	0.6492	0.9084	0.019*	
C72	0.43370 (16)	0.4953 (4)	0.88313 (13)	0.0216 (6)	
H72A	0.4649	0.4115	0.9022	0.032*	
H72B	0.4692	0.5750	0.8742	0.032*	
H72C	0.4014	0.4608	0.8463	0.032*	
C81	0.40454 (16)	0.3068 (3)	1.02043 (13)	0.0179 (6)	
H81A	0.3896	0.2828	1.0594	0.022*	
H81B	0.4361	0.4001	1.0251	0.022*	
C82	0.45590 (17)	0.1798 (3)	1.00273 (14)	0.0247 (7)	
H82A	0.4254	0.0866	0.9986	0.037*	
H82B	0.5025	0.1673	1.0334	0.037*	
H82C	0.4727	0.2043	0.9649	0.037*	
C121	0.25722 (17)	−0.1674 (3)	1.08425 (12)	0.0173 (6)	
H12A	0.2350	−0.2336	1.1121	0.021*	
H12B	0.2803	−0.0789	1.1068	0.021*	
C122	0.32288 (18)	−0.2513 (4)	1.05987 (14)	0.0259 (7)	
H12C	0.3011	−0.3423	1.0394	0.039*	
H12D	0.3646	−0.2786	1.0927	0.039*	
H12E	0.3448	−0.1868	1.0319	0.039*	
C131	0.08971 (17)	−0.3261 (3)	1.04001 (13)	0.0177 (6)	
H13A	0.0318	−0.3177	1.0377	0.021*	
H13B	0.1131	−0.3409	1.0822	0.021*	
C132	0.10840 (18)	−0.4624 (3)	1.00411 (13)	0.0215 (6)	
H13C	0.0829	−0.4508	0.9628	0.032*	
H13D	0.0886	−0.5532	1.0208	0.032*	
H13E	0.1657	−0.4704	1.0058	0.032*	
C171	−0.15641 (16)	−0.2129 (3)	0.87054 (14)	0.0209 (6)	
H17A	−0.2123	−0.1991	0.8522	0.025*	
H17B	−0.1546	−0.2262	0.9138	0.025*	
C172	−0.12383 (18)	−0.3538 (3)	0.84486 (14)	0.0236 (6)	
H17C	−0.1285	−0.3436	0.8017	0.035*	
H17D	−0.1540	−0.4414	0.8542	0.035*	
H17E	−0.0682	−0.3667	0.8623	0.035*	
C181	−0.19486 (16)	0.0360 (3)	0.76589 (13)	0.0220 (6)	
H18A	−0.2122	0.1412	0.7591	0.026*	
H18B	−0.2397	−0.0219	0.7765	0.026*	
C182	−0.17231 (19)	−0.0278 (4)	0.70848 (14)	0.0326 (8)	
H18C	−0.1273	0.0281	0.6982	0.049*	
H18D	−0.2173	−0.0186	0.6762	0.049*	
H18E	−0.1580	−0.1336	0.7143	0.049*	

### Atomic displacement parameters (Å^2^)

**Table:**

	*U*^11^	*U*^22^	*U*^33^	*U*^12^	*U*^13^	*U*^23^
Pt	0.00960 (5)	0.01056 (5)	0.01058 (5)	−0.00107 (4)	0.00043 (3)	−0.00166 (4)
N21	0.0124 (10)	0.0135 (10)	0.0103 (11)	0.0002 (9)	0.0003 (8)	−0.0025 (8)
N22	0.0128 (10)	0.0109 (11)	0.0112 (11)	−0.0009 (8)	0.0006 (8)	−0.0008 (8)
N23	0.0154 (11)	0.0126 (11)	0.0115 (11)	−0.0028 (9)	0.0019 (9)	−0.0025 (9)
N24	0.0121 (10)	0.0135 (11)	0.0152 (11)	−0.0010 (9)	0.0018 (9)	−0.0027 (9)
C1	0.0142 (12)	0.0153 (13)	0.0116 (13)	0.0043 (10)	−0.0004 (10)	−0.0027 (10)
C2	0.0163 (13)	0.0171 (13)	0.0110 (13)	0.0037 (11)	0.0004 (10)	−0.0028 (10)
C3	0.0144 (12)	0.0157 (13)	0.0105 (12)	0.0036 (10)	0.0022 (10)	−0.0002 (10)
C4	0.0135 (12)	0.0141 (13)	0.0098 (12)	0.0022 (10)	0.0025 (10)	−0.0034 (10)
C5	0.0129 (12)	0.0124 (12)	0.0117 (13)	−0.0020 (10)	0.0034 (10)	−0.0013 (10)
C6	0.0133 (12)	0.0118 (12)	0.0106 (12)	−0.0012 (10)	0.0019 (9)	−0.0021 (10)
C7	0.0119 (12)	0.0135 (13)	0.0114 (12)	−0.0009 (10)	0.0010 (10)	−0.0034 (10)
C8	0.0142 (12)	0.0131 (13)	0.0144 (13)	−0.0012 (10)	0.0005 (10)	−0.0015 (10)
C9	0.0119 (12)	0.0123 (12)	0.0135 (13)	−0.0016 (10)	0.0002 (10)	−0.0027 (10)
C10	0.0139 (12)	0.0151 (13)	0.0116 (13)	−0.0008 (10)	−0.0018 (10)	−0.0005 (10)
C11	0.0143 (12)	0.0149 (13)	0.0116 (13)	0.0020 (10)	0.0024 (10)	−0.0012 (10)
C12	0.0196 (13)	0.0137 (13)	0.0114 (13)	0.0000 (10)	0.0053 (10)	−0.0011 (10)
C13	0.0182 (13)	0.0135 (13)	0.0121 (13)	−0.0019 (10)	0.0056 (10)	−0.0017 (10)
C14	0.0162 (12)	0.0112 (12)	0.0132 (13)	−0.0024 (10)	0.0051 (10)	−0.0032 (10)
C15	0.0183 (13)	0.0122 (13)	0.0177 (14)	−0.0036 (10)	0.0077 (11)	−0.0031 (10)
C16	0.0121 (12)	0.0151 (13)	0.0175 (14)	−0.0036 (10)	0.0048 (10)	−0.0062 (10)
C17	0.0110 (12)	0.0208 (14)	0.0197 (14)	−0.0028 (11)	0.0053 (10)	−0.0084 (12)
C18	0.0105 (12)	0.0194 (14)	0.0207 (14)	−0.0007 (10)	0.0015 (11)	−0.0083 (11)
C19	0.0127 (12)	0.0167 (13)	0.0165 (14)	0.0016 (10)	0.0011 (10)	−0.0063 (11)
C20	0.0124 (12)	0.0187 (13)	0.0148 (13)	0.0034 (11)	−0.0029 (10)	−0.0042 (11)
C21	0.0189 (14)	0.0194 (14)	0.0152 (14)	0.0020 (11)	−0.0026 (11)	−0.0003 (11)
C22	0.0280 (16)	0.0390 (19)	0.0177 (15)	0.0100 (14)	−0.0080 (12)	−0.0062 (14)
C31	0.0169 (13)	0.0149 (14)	0.0143 (13)	0.0017 (10)	0.0014 (10)	0.0011 (10)
C32	0.0214 (14)	0.0153 (13)	0.0236 (15)	0.0024 (11)	0.0052 (12)	0.0001 (11)
C51	0.0118 (12)	0.0133 (12)	0.0119 (13)	−0.0003 (10)	−0.0005 (10)	0.0021 (10)
C52	0.0155 (13)	0.0159 (14)	0.0137 (13)	0.0007 (10)	0.0030 (10)	0.0012 (10)
C53	0.0215 (14)	0.0151 (13)	0.0200 (14)	−0.0007 (11)	0.0010 (11)	−0.0011 (11)
C54	0.0186 (14)	0.0164 (14)	0.0250 (15)	−0.0034 (11)	0.0034 (11)	0.0068 (12)
C55	0.0171 (13)	0.0229 (15)	0.0185 (14)	−0.0006 (11)	0.0049 (11)	0.0070 (11)
C56	0.0175 (13)	0.0170 (13)	0.0101 (13)	0.0012 (11)	0.0004 (10)	0.0008 (10)
C71	0.0145 (12)	0.0179 (14)	0.0155 (13)	−0.0050 (11)	0.0002 (10)	0.0024 (11)
C72	0.0164 (14)	0.0274 (16)	0.0210 (15)	0.0004 (12)	0.0031 (11)	0.0040 (12)
C81	0.0151 (13)	0.0171 (13)	0.0191 (14)	−0.0027 (11)	−0.0050 (11)	0.0029 (11)
C82	0.0167 (14)	0.0257 (16)	0.0309 (17)	0.0021 (12)	0.0014 (12)	0.0079 (13)
C121	0.0220 (14)	0.0155 (14)	0.0138 (13)	−0.0012 (11)	0.0008 (11)	0.0018 (10)
C122	0.0235 (15)	0.0250 (15)	0.0284 (17)	0.0059 (13)	0.0021 (13)	0.0009 (13)
C131	0.0214 (14)	0.0160 (14)	0.0166 (14)	−0.0025 (11)	0.0064 (11)	0.0010 (11)
C132	0.0282 (16)	0.0142 (14)	0.0239 (16)	−0.0053 (12)	0.0095 (12)	−0.0022 (12)
C171	0.0136 (13)	0.0224 (15)	0.0269 (16)	−0.0045 (11)	0.0040 (11)	−0.0041 (12)
C172	0.0249 (15)	0.0175 (14)	0.0282 (17)	−0.0064 (12)	0.0035 (13)	−0.0046 (12)
C181	0.0139 (13)	0.0223 (15)	0.0267 (16)	−0.0011 (11)	−0.0054 (11)	−0.0046 (12)
C182	0.0258 (16)	0.041 (2)	0.0263 (18)	0.0055 (15)	−0.0095 (13)	−0.0127 (15)

### Geometric parameters (Å, °)

**Table:**

Pt—N21	2.019 (2)	C31—H31B	0.9900
Pt—N22	2.020 (2)	C32—H32A	0.9800
Pt—N23	2.023 (2)	C32—H32B	0.9800
Pt—N24	2.025 (2)	C32—H32C	0.9800
N21—C1	1.378 (3)	C51—C52	1.394 (4)
N21—C4	1.390 (3)	C51—C56	1.400 (4)
N22—C9	1.377 (3)	C52—C53	1.391 (4)
N22—C6	1.388 (3)	C52—H52	0.9500
N23—C14	1.378 (3)	C53—C54	1.388 (4)
N23—C11	1.382 (3)	C53—H53	0.9500
N24—C16	1.374 (3)	C54—C55	1.392 (4)
N24—C19	1.376 (3)	C54—H54	0.9500
C1—C20	1.387 (4)	C55—C56	1.389 (4)
C1—C2	1.438 (4)	C55—H55	0.9500
C2—C3	1.359 (4)	C56—H56	0.9500
C2—C21	1.502 (4)	C71—C72	1.538 (4)
C3—C4	1.469 (4)	C71—H71A	0.9900
C3—C31	1.514 (4)	C71—H71B	0.9900
C4—C5	1.400 (4)	C72—H72A	0.9800
C5—C6	1.404 (4)	C72—H72B	0.9800
C5—C51	1.496 (4)	C72—H72C	0.9800
C6—C7	1.472 (3)	C81—C82	1.531 (4)
C7—C8	1.364 (4)	C81—H81A	0.9900
C7—C71	1.515 (4)	C81—H81B	0.9900
C8—C9	1.438 (4)	C82—H82A	0.9800
C8—C81	1.498 (4)	C82—H82B	0.9800
C9—C10	1.384 (4)	C82—H82C	0.9800
C10—C11	1.375 (4)	C121—C122	1.532 (4)
C10—H10	0.9500	C121—H12A	0.9900
C11—C12	1.450 (4)	C121—H12B	0.9900
C12—C13	1.360 (4)	C122—H12C	0.9800
C12—C121	1.496 (4)	C122—H12D	0.9800
C13—C14	1.447 (4)	C122—H12E	0.9800
C13—C131	1.501 (4)	C131—C132	1.531 (4)
C14—C15	1.375 (4)	C131—H13A	0.9900
C15—C16	1.385 (4)	C131—H13B	0.9900
C15—H15	0.9500	C132—H13C	0.9800
C16—C17	1.451 (4)	C132—H13D	0.9800
C17—C18	1.361 (4)	C132—H13E	0.9800
C17—C171	1.499 (4)	C171—C172	1.532 (4)
C18—C19	1.448 (4)	C171—H17A	0.9900
C18—C181	1.508 (4)	C171—H17B	0.9900
C19—C20	1.377 (4)	C172—H17C	0.9800
C20—H20	0.9500	C172—H17D	0.9800
C21—C22	1.529 (4)	C172—H17E	0.9800
C21—H21A	0.9900	C181—C182	1.536 (4)
C21—H21B	0.9900	C181—H18A	0.9900
C22—H22A	0.9800	C181—H18B	0.9900
C22—H22B	0.9800	C182—H18C	0.9800
C22—H22C	0.9800	C182—H18D	0.9800
C31—C32	1.534 (4)	C182—H18E	0.9800
C31—H31A	0.9900		
			
N21—Pt—N22	88.69 (9)	C31—C32—H32A	109.5
N21—Pt—N23	179.84 (9)	C31—C32—H32B	109.5
N22—Pt—N23	91.47 (9)	H32A—C32—H32B	109.5
N21—Pt—N24	91.55 (9)	C31—C32—H32C	109.5
N22—Pt—N24	178.79 (9)	H32A—C32—H32C	109.5
N23—Pt—N24	88.29 (9)	H32B—C32—H32C	109.5
C1—N21—C4	106.5 (2)	C52—C51—C56	119.8 (2)
C1—N21—Pt	124.68 (18)	C52—C51—C5	119.7 (2)
C4—N21—Pt	128.82 (17)	C56—C51—C5	120.5 (2)
C9—N22—C6	106.6 (2)	C53—C52—C51	119.9 (3)
C9—N22—Pt	124.63 (17)	C53—C52—H52	120.0
C6—N22—Pt	128.76 (17)	C51—C52—H52	120.0
C14—N23—C11	105.5 (2)	C54—C53—C52	120.0 (3)
C14—N23—Pt	128.36 (17)	C54—C53—H53	120.0
C11—N23—Pt	126.12 (18)	C52—C53—H53	120.0
C16—N24—C19	106.0 (2)	C53—C54—C55	120.4 (3)
C16—N24—Pt	128.04 (18)	C53—C54—H54	119.8
C19—N24—Pt	125.93 (18)	C55—C54—H54	119.8
N21—C1—C20	126.0 (3)	C56—C55—C54	119.7 (3)
N21—C1—C2	110.2 (2)	C56—C55—H55	120.2
C20—C1—C2	123.8 (2)	C54—C55—H55	120.2
C3—C2—C1	107.7 (2)	C55—C56—C51	120.1 (3)
C3—C2—C21	127.6 (3)	C55—C56—H56	119.9
C1—C2—C21	124.7 (2)	C51—C56—H56	119.9
C2—C3—C4	106.6 (2)	C7—C71—C72	111.9 (2)
C2—C3—C31	121.8 (2)	C7—C71—H71A	109.2
C4—C3—C31	131.6 (2)	C72—C71—H71A	109.2
N21—C4—C5	124.0 (2)	C7—C71—H71B	109.2
N21—C4—C3	109.0 (2)	C72—C71—H71B	109.2
C5—C4—C3	126.9 (2)	H71A—C71—H71B	107.9
C4—C5—C6	125.6 (2)	C71—C72—H72A	109.5
C4—C5—C51	117.9 (2)	C71—C72—H72B	109.5
C6—C5—C51	116.5 (2)	H72A—C72—H72B	109.5
N22—C6—C5	124.0 (2)	C71—C72—H72C	109.5
N22—C6—C7	108.9 (2)	H72A—C72—H72C	109.5
C5—C6—C7	127.1 (2)	H72B—C72—H72C	109.5
C8—C7—C6	106.6 (2)	C8—C81—C82	113.7 (2)
C8—C7—C71	121.8 (2)	C8—C81—H81A	108.8
C6—C7—C71	131.5 (2)	C82—C81—H81A	108.8
C7—C8—C9	107.5 (2)	C8—C81—H81B	108.8
C7—C8—C81	128.3 (2)	C82—C81—H81B	108.8
C9—C8—C81	124.2 (2)	H81A—C81—H81B	107.7
N22—C9—C10	126.3 (2)	C81—C82—H82A	109.5
N22—C9—C8	110.4 (2)	C81—C82—H82B	109.5
C10—C9—C8	123.2 (2)	H82A—C82—H82B	109.5
C11—C10—C9	127.0 (2)	C81—C82—H82C	109.5
C11—C10—H10	116.5	H82A—C82—H82C	109.5
C9—C10—H10	116.5	H82B—C82—H82C	109.5
C10—C11—N23	124.4 (2)	C12—C121—C122	113.2 (2)
C10—C11—C12	125.1 (2)	C12—C121—H12A	108.9
N23—C11—C12	110.5 (2)	C122—C121—H12A	108.9
C13—C12—C11	106.5 (2)	C12—C121—H12B	108.9
C13—C12—C121	128.5 (2)	C122—C121—H12B	108.9
C11—C12—C121	125.0 (2)	H12A—C121—H12B	107.8
C12—C13—C14	107.1 (2)	C121—C122—H12C	109.5
C12—C13—C131	128.1 (3)	C121—C122—H12D	109.5
C14—C13—C131	124.8 (2)	H12C—C122—H12D	109.5
C15—C14—N23	124.8 (2)	C121—C122—H12E	109.5
C15—C14—C13	124.8 (2)	H12C—C122—H12E	109.5
N23—C14—C13	110.4 (2)	H12D—C122—H12E	109.5
C14—C15—C16	125.4 (3)	C13—C131—C132	112.3 (2)
C14—C15—H15	117.3	C13—C131—H13A	109.1
C16—C15—H15	117.3	C132—C131—H13A	109.1
N24—C16—C15	125.1 (2)	C13—C131—H13B	109.1
N24—C16—C17	110.3 (2)	C132—C131—H13B	109.1
C15—C16—C17	124.6 (3)	H13A—C131—H13B	107.9
C18—C17—C16	106.6 (2)	C131—C132—H13C	109.5
C18—C17—C171	129.3 (3)	C131—C132—H13D	109.5
C16—C17—C171	124.1 (3)	H13C—C132—H13D	109.5
C17—C18—C19	106.9 (2)	C131—C132—H13E	109.5
C17—C18—C181	128.7 (3)	H13C—C132—H13E	109.5
C19—C18—C181	124.2 (3)	H13D—C132—H13E	109.5
N24—C19—C20	124.6 (2)	C17—C171—C172	111.9 (2)
N24—C19—C18	110.2 (2)	C17—C171—H17A	109.2
C20—C19—C18	125.1 (3)	C172—C171—H17A	109.2
C19—C20—C1	127.1 (3)	C17—C171—H17B	109.2
C19—C20—H20	116.5	C172—C171—H17B	109.2
C1—C20—H20	116.5	H17A—C171—H17B	107.9
C2—C21—C22	112.1 (2)	C171—C172—H17C	109.5
C2—C21—H21A	109.2	C171—C172—H17D	109.5
C22—C21—H21A	109.2	H17C—C172—H17D	109.5
C2—C21—H21B	109.2	C171—C172—H17E	109.5
C22—C21—H21B	109.2	H17C—C172—H17E	109.5
H21A—C21—H21B	107.9	H17D—C172—H17E	109.5
C21—C22—H22A	109.5	C18—C181—C182	111.8 (2)
C21—C22—H22B	109.5	C18—C181—H18A	109.3
H22A—C22—H22B	109.5	C182—C181—H18A	109.3
C21—C22—H22C	109.5	C18—C181—H18B	109.3
H22A—C22—H22C	109.5	C182—C181—H18B	109.3
H22B—C22—H22C	109.5	H18A—C181—H18B	107.9
C3—C31—C32	112.4 (2)	C181—C182—H18C	109.5
C3—C31—H31A	109.1	C181—C182—H18D	109.5
C32—C31—H31A	109.1	H18C—C182—H18D	109.5
C3—C31—H31B	109.1	C181—C182—H18E	109.5
C32—C31—H31B	109.1	H18C—C182—H18E	109.5
H31A—C31—H31B	107.9	H18D—C182—H18E	109.5
			
N22—Pt—N21—C1	176.0 (2)	C10—C11—C12—C13	179.9 (3)
N24—Pt—N21—C1	−2.8 (2)	N23—C11—C12—C13	−0.1 (3)
N22—Pt—N21—C4	−3.1 (2)	C10—C11—C12—C121	−1.5 (4)
N24—Pt—N21—C4	178.1 (2)	N23—C11—C12—C121	178.4 (2)
N21—Pt—N22—C9	179.2 (2)	C11—C12—C13—C14	−0.2 (3)
N23—Pt—N22—C9	−0.8 (2)	C121—C12—C13—C14	−178.6 (3)
N21—Pt—N22—C6	−1.1 (2)	C11—C12—C13—C131	178.1 (3)
N23—Pt—N22—C6	179.0 (2)	C121—C12—C13—C131	−0.4 (5)
N22—Pt—N23—C14	−179.3 (2)	C11—N23—C14—C15	179.3 (3)
N24—Pt—N23—C14	−0.5 (2)	Pt—N23—C14—C15	−0.5 (4)
N22—Pt—N23—C11	1.0 (2)	C11—N23—C14—C13	−0.4 (3)
N24—Pt—N23—C11	179.8 (2)	Pt—N23—C14—C13	179.77 (17)
N21—Pt—N24—C16	−179.2 (2)	C12—C13—C14—C15	−179.4 (3)
N23—Pt—N24—C16	0.7 (2)	C131—C13—C14—C15	2.3 (4)
N21—Pt—N24—C19	3.9 (2)	C12—C13—C14—N23	0.4 (3)
N23—Pt—N24—C19	−176.2 (2)	C131—C13—C14—N23	−178.0 (2)
C4—N21—C1—C20	−179.6 (3)	N23—C14—C15—C16	1.5 (4)
Pt—N21—C1—C20	1.1 (4)	C13—C14—C15—C16	−178.8 (3)
C4—N21—C1—C2	−0.3 (3)	C19—N24—C16—C15	177.3 (3)
Pt—N21—C1—C2	−179.58 (17)	Pt—N24—C16—C15	−0.1 (4)
N21—C1—C2—C3	−1.0 (3)	C19—N24—C16—C17	−0.1 (3)
C20—C1—C2—C3	178.3 (3)	Pt—N24—C16—C17	−177.57 (17)
N21—C1—C2—C21	176.1 (2)	C14—C15—C16—N24	−1.2 (4)
C20—C1—C2—C21	−4.6 (4)	C14—C15—C16—C17	175.9 (3)
C1—C2—C3—C4	1.8 (3)	N24—C16—C17—C18	0.8 (3)
C21—C2—C3—C4	−175.2 (3)	C15—C16—C17—C18	−176.7 (3)
C1—C2—C3—C31	−177.8 (2)	N24—C16—C17—C171	177.1 (2)
C21—C2—C3—C31	5.3 (4)	C15—C16—C17—C171	−0.3 (4)
C1—N21—C4—C5	−174.8 (2)	C16—C17—C18—C19	−1.0 (3)
Pt—N21—C4—C5	4.4 (4)	C171—C17—C18—C19	−177.1 (3)
C1—N21—C4—C3	1.4 (3)	C16—C17—C18—C181	174.1 (3)
Pt—N21—C4—C3	−179.37 (17)	C171—C17—C18—C181	−2.1 (5)
C2—C3—C4—N21	−2.0 (3)	C16—N24—C19—C20	179.2 (3)
C31—C3—C4—N21	177.4 (2)	Pt—N24—C19—C20	−3.3 (4)
C2—C3—C4—C5	174.1 (3)	C16—N24—C19—C18	−0.5 (3)
C31—C3—C4—C5	−6.5 (4)	Pt—N24—C19—C18	177.00 (17)
N21—C4—C5—C6	−0.7 (4)	C17—C18—C19—N24	1.0 (3)
C3—C4—C5—C6	−176.3 (3)	C181—C18—C19—N24	−174.4 (2)
N21—C4—C5—C51	−179.6 (2)	C17—C18—C19—C20	−178.7 (3)
C3—C4—C5—C51	4.9 (4)	C181—C18—C19—C20	5.9 (4)
C9—N22—C6—C5	−175.9 (2)	N24—C19—C20—C1	0.3 (5)
Pt—N22—C6—C5	4.3 (4)	C18—C19—C20—C1	179.9 (3)
C9—N22—C6—C7	1.8 (3)	N21—C1—C20—C19	0.9 (5)
Pt—N22—C6—C7	−177.95 (17)	C2—C1—C20—C19	−178.3 (3)
C4—C5—C6—N22	−3.7 (4)	C3—C2—C21—C22	92.3 (3)
C51—C5—C6—N22	175.2 (2)	C1—C2—C21—C22	−84.2 (3)
C4—C5—C6—C7	179.0 (2)	C2—C3—C31—C32	86.4 (3)
C51—C5—C6—C7	−2.1 (4)	C4—C3—C31—C32	−93.0 (3)
N22—C6—C7—C8	−2.1 (3)	C4—C5—C51—C52	89.9 (3)
C5—C6—C7—C8	175.5 (3)	C6—C5—C51—C52	−89.1 (3)
N22—C6—C7—C71	173.7 (3)	C4—C5—C51—C56	−92.0 (3)
C5—C6—C7—C71	−8.7 (5)	C6—C5—C51—C56	89.0 (3)
C6—C7—C8—C9	1.6 (3)	C56—C51—C52—C53	−0.2 (4)
C71—C7—C8—C9	−174.7 (2)	C5—C51—C52—C53	177.8 (2)
C6—C7—C8—C81	179.0 (3)	C51—C52—C53—C54	−0.3 (4)
C71—C7—C8—C81	2.7 (4)	C52—C53—C54—C55	0.4 (4)
C6—N22—C9—C10	179.7 (3)	C53—C54—C55—C56	0.1 (4)
Pt—N22—C9—C10	−0.5 (4)	C54—C55—C56—C51	−0.6 (4)
C6—N22—C9—C8	−0.9 (3)	C52—C51—C56—C55	0.7 (4)
Pt—N22—C9—C8	178.91 (17)	C5—C51—C56—C55	−177.3 (2)
C7—C8—C9—N22	−0.5 (3)	C8—C7—C71—C72	85.1 (3)
C81—C8—C9—N22	−178.1 (2)	C6—C7—C71—C72	−90.2 (3)
C7—C8—C9—C10	179.0 (3)	C7—C8—C81—C82	−101.4 (3)
C81—C8—C9—C10	1.4 (4)	C9—C8—C81—C82	75.6 (3)
N22—C9—C10—C11	2.2 (5)	C13—C12—C121—C122	98.1 (3)
C8—C9—C10—C11	−177.2 (3)	C11—C12—C121—C122	−80.1 (3)
C9—C10—C11—N23	−1.9 (4)	C12—C13—C131—C132	−92.1 (3)
C9—C10—C11—C12	178.0 (3)	C14—C13—C131—C132	85.8 (3)
C14—N23—C11—C10	−179.7 (2)	C18—C17—C171—C172	98.0 (3)
Pt—N23—C11—C10	0.1 (4)	C16—C17—C171—C172	−77.5 (3)
C14—N23—C11—C12	0.3 (3)	C17—C18—C181—C182	−96.4 (4)
Pt—N23—C11—C12	−179.86 (17)	C19—C18—C181—C182	77.9 (4)
